# A Rare Case of Concurrent 2q34q36 Duplication and 2q37 Deletion in a Neonate with Syndromic Features

**DOI:** 10.3390/genes14122194

**Published:** 2023-12-10

**Authors:** Francesco Nicola Riviello, Alessia Daponte, Emanuela Ponzi, Romina Ficarella, Paola Orsini, Roberta Bucci, Mario Ventura, Francesca Antonacci, Claudia Rita Catacchio, Mattia Gentile

**Affiliations:** 1 U.O.C. Laboratorio di Genetica Medica, PO Di Venere—ASL Bari, 70012 Bari, Italy; francesco.riviello@asl.bari.it (F.N.R.); emanuela.ponzi@asl.bari.it (E.P.); romina.ficarella@asl.bari.it (R.F.); paola.orsini@asl.bari.it (P.O.); bucciroberta23@gmail.com (R.B.); 2 Dipartimento di Bioscienze, Biotecnologie e Ambiente, Università degli Studi di Bari “Aldo Moro”, 70125 Bari, Italy; alessia.daponte@uniba.it (A.D.); mario.ventura@uniba.it (M.V.); francesca.antonacci@uniba.it (F.A.)

**Keywords:** microdeletion, microduplication, structural rearrangements, chromosome abnormalities

## Abstract

Large-scale genomic structural variations can have significant clinical implications, depending on the specific altered genomic region. Briefly, 2q37 microdeletion syndrome is a prevalent subtelomeric deletion disorder characterized by variable-sized deletions. Affected patients exhibit a wide range of clinical manifestations, including short stature, facial dysmorphism, and features of autism spectrum disorder, among others. Conversely, isolated duplications of proximal chromosome 2q are rare and lack a distinct phenotype. In this report, we provide an extensive molecular analysis of a 15-day-old newborn referred for syndromic features. Our analysis reveals an 8.5 Mb microdeletion at 2q37.1, which extends to the telomere, in conjunction with an 8.6 Mb interstitial microduplication at 2q34q36.1. Our findings underscore the prominence of 2q37 terminal deletions as commonly reported genomic anomalies. We compare our patient’s phenotype with previously reported cases in the literature to contribute to a more refined classification of 2q37 microdeletion syndrome and assess the potential impact of 2q34q36.1 microduplication. We also investigate multiple hypotheses to clarify the genetic mechanisms responsible for the observed genomic rearrangement.

## 1. Introduction

Structural chromosomal rearrangements involving large regions of one- to several-megabase pairs arise through a variety of mechanisms often associated with particular features of genomic architecture, which can trigger genetic instability [1]. Chromosome abnormalities can have significant implications, particularly when they affect the balance of genes that can lead to the development of various diseases.

Chromosome imbalances affecting the long arm of chromosome 2 result in a variety of distinct clinical conditions. For example, 2q37 microdeletion syndrome, alternatively known as Albright hereditary osteodystrophy-like syndrome or brachydactyly-intellectual disability syndrome, is a rare genetic disorder resulting from a variable-sized deletion in the long (q) arm of chromosome 2 [2,3,4]. The syndrome is characterized by a broad spectrum of clinical findings: the most common phenotypic features include mild to moderate developmental delay/intellectual disability (ID), brachymetaphalangy of digits 3–5 (brachydactyly type E), short stature, obesity, hypotonia in infancy, abnormal behavior with autism spectrum disorder, joint hypermobility, and scoliosis. Most individuals with 2q37 deletion syndrome have a typical dysmorphic face: broad or rounded facies; frontal bossing; midface hypoplasia; thin, arched eyebrows with deep-set eyes; upslanting palpebral fissures; prominent columella; and minor ear defects. In 20–30% of cases, visceral malformations are also present: congenital heart disease (mostly septal defects), gastrointestinal or genitourinary anomalies, central nervous system malformations, renal anomalies, and Wilms tumors. Rarely, patients may have associated seizures and hyperactivity with attention deficit disorder [3].

On the other hand, duplications of proximal chromosome 2q are rare, and no specific associated syndrome has been identified. The majority of trisomy 2q cases arise from parental rearrangements, often accompanied by a deletion of another chromosomal segment, which hampers phenotypic delineation. Pure trisomy 2q is uncommon, and some reports compare patients with varying sizes of affected chromosomal segments [5]. Consequently, attributing abnormalities related to trisomy 2q poses a significant challenge [6]. A distinction is commonly drawn between a more proximal and a distal trisomy 2q phenotype [7]. Duplications proximal to 2q33 tend to result in a more severe phenotype characterized by major malformations and substantial growth and developmental retardation, while duplications distal to 2q33 appear to be milder [8,9].

Terminal deletions with concomitant duplications are complex chromosomal rearrangements characterized by the loss of a chromosomal segment at one end (terminal deletion) and the duplication of another segment on the same arm, and have been mostly reported in the context of inv-dup del syndromes [10]. To the best of our knowledge, only a few subjects with inv-dup del involving the long arm of chromosome 2 have been documented [11,12,13]. 

Here, we report on the clinical, cytogenetic, and molecular analysis of a 15-day-old newborn referred for syndromic features. Through a combination of SNP array and fluorescence in situ hybridization (FISH), we demonstrate that the patient carries a de novo 2q37.1q37.3 microdeletion and an unusual interstitial microduplication at 2q34q36.1 in a direct orientation. The parents are phenotypically normal and do not carry any inversion of the region, which might be implicated in the rearrangement onset. Furthermore, we provide a comparative analysis of the phenotypic and clinical characteristics of our patient with those of other relevant cases described in the literature.

## 2. Materials and Methods

Patient. The patient was referred to our attention from Neonatology of Di Venere Hospital for a suspected syndromic condition. The child was evaluated by a neonatologist, and laboratory workup was performed for newborn screenings of SMA, cystic fibrosis, and inborn errors of metabolism. Written informed consent was obtained from the parents for carrying out genetic tests (Decipher ID: 522623).

Classical Cytogenetic Analysis. Conventional cytogenetic analysis was performed, according to standard methods on lymphocytes from phytohemaglutinin-stimulated peripheral blood cultures, on the patient and his parents. Chromosome spreads were processed for QFQ banding [14].

Fluorescence In Situ Hybridization. Metaphase spreads and interphase nuclei were obtained from phytohemagglutinin (PHA)-stimulated whole-blood cultures. For the FISH experiments, 9 human BAC clones (Table S1) were directly labeled through nick-translation with Cy3-dUTP, Cy5-dUTP, and fluorescein-dUTP (Enzo), following the protocol outlined by Lichter et al. [15], with slight modifications. In brief, 300 ng of the labeled probe was utilized for the FISH experiments, and hybridization occurred at 37 °C in 2 × SSC, 50% (*v*/*v*) formamide, 10% (*w*/*v*) dextran sulphate, 3 μg of Cot-1 DNA, and 3 mg of sonicated salmon sperm DNA, in a 10 μL volume. Posthybridization washing was conducted under high-stringency conditions at 60 °C in 0.1 × SSC (three times). Simultaneous DAPI staining was applied to both nuclei and chromosome metaphases. Digital images were captured using a Leica DMRXA2 epifluorescence microscope equipped with a cooled CCD camera (Princeton Instruments). DAPI, Cy3, Cy5, and fluorescein fluorescence signals, detected through specific filters, were individually recorded as grayscale images. 

SNP Array. The patient and both parents were analyzed via Array-CGH following the manufacturer’s instructions. The procedure utilized CytoSNP-850K (Illumina, San Diego, CA, USA), which contains over 850,000 markers. Approximately 200 ng of genomic DNA was used to genotype each sample. Samples were processed according to the Infinium HD assay manual. Briefly, each sample was whole-genome-amplified, fragmented, precipitated, and resuspended in an appropriate hybridization buffer. Denatured samples were hybridized on CytoSNP-850K v1.3 BeadChip for a minimum of 16 h at 48 °C. After the completion of the assay, BeadChips were scanned with dual-color NextSeq550 (Illumina, San Diego, CA, USA). Image intensities were extracted and analyzed using Illumina’s BlueFuse Multi Software Edition 4.5.

## 3. Results

Clinical Report. The proband was born to Indian, nonconsanguineous, phenotypically normal parents. Family history was not remarkable for ID and epilepsy in the paternal and maternal lineage.

**Figure 1:**
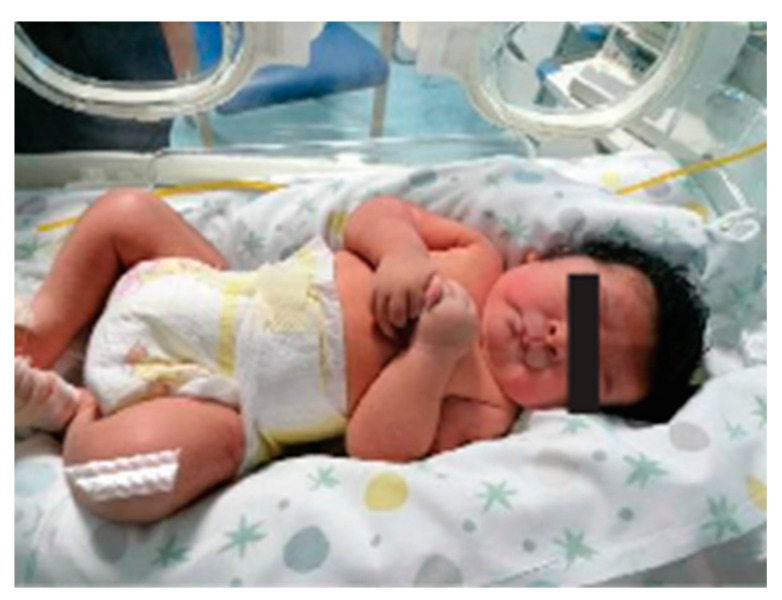
Appearance of proband at 15 days of life.

He was born at 39 weeks via cesarean section due to maternal hypertension and gestational diabetes. Oligohydramnios was reported. His birth weight was 3910 g (>97th centile-LGA), and his Apgar score was 9-9, at 1° and 5° minutes, respectively. The patient was referred at the age of 15 days of life due to syndromic features (Figure 1). Physical examination showed thick hair, bilateral epicanthus, anteverted nares, long philtrum, pterygium colli, normally shaped hands with a single palmar fold, tapered fingers, long nails, lower incisors implanted on the mobile gum line, and hypertrichosis of the lower limbs. Brain and heart ultrasound examinations were normal. 

At the age of 6 months, he had a severe respiratory infection that required hospitalization and treatment with aerosols and antibiotics. At the last clinical examination (age 9 months), the auxological parameters were as follows: weight, 10 kg (75th centile); height, 73 cm (50–75th centile); and head circumference, 47 cm (75–90th centile). He suffered from recurrent rhinitis. Early signs of developmental delay, significant hypotonia, and joint laxity were present. He did not have good head control and was unable to sit independently. Abdominal and renal ultrasound, echocardiography, and ophthalmological and ORL examinations were all normal. Complete blood count, liver and renal function tests, blood glucose, IgA, IgG, and IgM levels were all within normal limits. 

**Figure 2:**
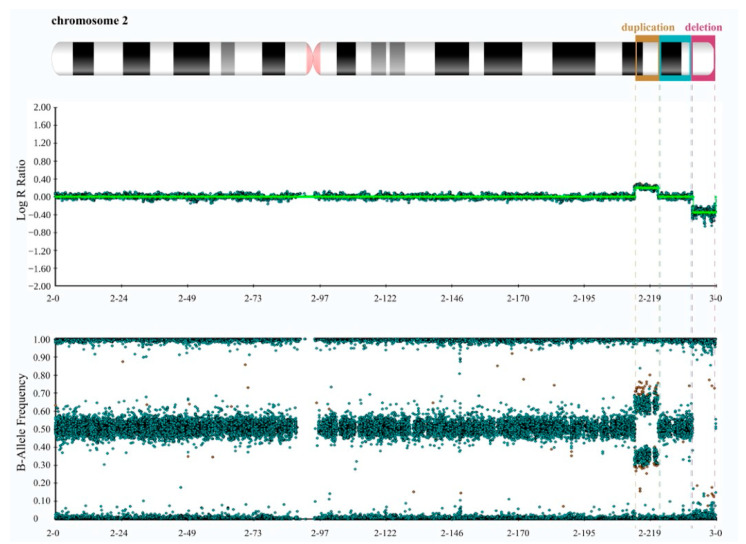
High-resolution SNP array data of the proband reveal a microduplication at 2q34q36.1 and a microdeletion at 2q37.1. The top panel displays log R ratios (LRR) across the chromosome. LRR is a normalized measure of signal intensity used to assess the copy number alteration of a given region. It is a base-2 log of the observed intensity for a given SNP in the investigated sample over the expected intensity for that SNP, calculated in a normal diploid population. Zero indicates a typical, diploid copy number. Elevated and reduced values signify regions that have experienced copy number gains and losses, respectively. The lower panel exhibits B allele frequency (BAF) values. BAF is a measure of the contribution of the genotyped alleles to the intensity found at that locus and shows the proportion of alleles present in the samples. For each SNP, BAF values of 1, 0.5, and 0 are associated with BB, AB, and AA genotypes, respectively. By combining LRR and BAF values, more confidence in calls is obtained, and copy number alterations, loss of heterozygosity (LOH) regions, unbalanced aberrations, and mosaicisms can be detected. The panels were extracted using Illumina’s BlueFuse Multi Software (Edition 4.5) and depict the characteristic spread pattern of probes across the chromosome, taking into account the presence of minimal background noise.

Cytogenetic and Molecular Analyses. We initially conducted the karyotype test on both the patient and his parents, revealing the absence of significant chromosome rearrangements in any of them (Figure S1). However, given the patient’s observed phenotype and the low resolution of karyotyping, we proceeded with a genetic evaluation of the patient and both parents by performing high-resolution SNP array hybridization. It revealed, exclusively in the patient, an 8.6 Mb interstitial duplication at 2q34q36.1 (chr2:212391040-220970912, GRCh38/hg38) and an 8.5 Mb deletion at 2q37.1 (chr2:233621539-242042818, GRCh38/hg38) that extended to the telomere (Figure 2 and Figure S2). 

**Figure 3:**
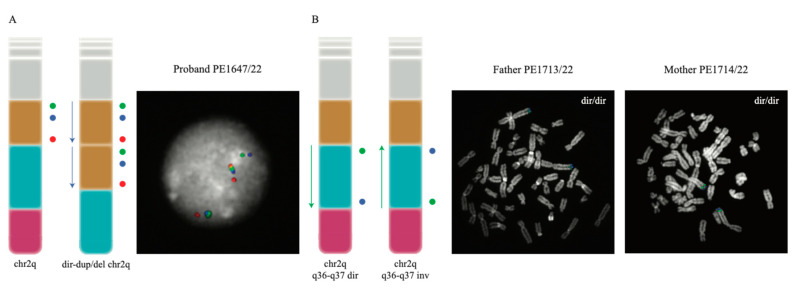
FISH experiments performed to investigate the orientation of the investigated regions. (**A**) illustrates the probe distribution (as colored circles) both in the normal 2q chromosome and in the rearranged chromosome found in the proband (**left**); FISH results for one of the analyzed interphase nuclei of the proband are also shown (**right**). (**B**) The design and the results of the FISH experiment designed to test the orientation of the euploid region; the probe order (represented as colored circles) of the direct and inverted euploid region (**left**) and the experiment’s results on one of the analyzed metaphases for each parent of the proband (**right**) are presented. In both panels, the chr2q ideograms show the duplicated region in yellow, the euploid region in sky blue and the deleted one in pink.

Since the SNP array analysis can only provide information regarding unbalanced rearrangements, such as duplications and deletions, without providing details about the location of the duplicated copy or the directionality of the region involved in the rearrangement, we proceeded with the fine characterization of the rearrangement with a molecular cytogenetics approach. We performed metaphase FISH experiments on the proband and confirmed the duplication and the deletion map on the same chromosome (Exp1_DupDel in Table S1). We then performed interphase FISH experiments to evaluate the orientation of the two copies of the duplicated region (Exp2_Copy1 and Exp3_Copy2 in Table S1) and of the euchromatic region in between the duplication and the deletion (Exp4_Eu in Table S1). Both Exp2_Copy1 and Exp3_Copy2 proved that both copies of the region were in a direct orientation (Figure 3A). Exp4_Eu also showed the euploid region to be in a direct orientation in all the analyzed nuclei (Table S1). 

Duplication and deletion in an offspring are often caused by non-allelic homologous recombination (NAHR) events occurring when the duplicated or euchromatic region is inverted in one of the two parents. For this reason and to understand the mechanism leading to the rearrangement in the patient, we studied the region in the parents by performing Exp4_Eu, Exp5_Dup, and Exp6_Del FISH experiments. Both parents resulted in having the three regions in a direct orientation (Figure 3B and Table S1). 

**Figure 4:**
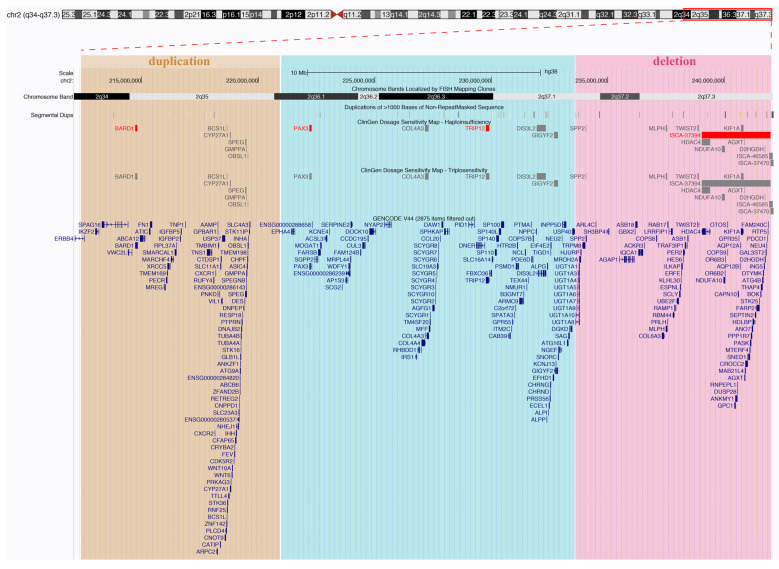
A UCSC Genome Browser representation illustrating the rearranged regions in our patient, featuring tracks from ClinGen dosage sensitivity and GENCODE. ClinGen tracks highlight red entries with a haploinsufficiency score of 3 (indicating sufficient evidence for dosage pathogenicity), while grey entries represent other evidence scores or those not yet evaluated (refer to Table S2 for additional details).

We investigated dosage sensitivity data [16,17,18] for genes within the rearranged regions in our patient and observed that genes identified within the duplication did not exhibit evidence supporting triplosensitivity or were not evaluated. Conversely, five genes within the deletion revealed an autosomal recessive haplotype, and one gene displayed limited evidence for dosage pathogenicity (Figure 4 and Table S2).

Additionally, we delved into the annotated pathogenicity of genes using the OMIM database (https://omim.org/, accessed on 28 November 2023). Our findings indicate that within the duplication, 76 genes are associated with 89 phenotypes (Table S3), while within the deletion, 55 genes are associated with 69 phenotypes (Table S4). Notably, ten genes within the deletion were not reported as being associated with any clinical phenotype (Table S5).

## 4. Discussion

Molecular characterization using the SNP array and FISH analysis on a patient with a de novo 2q37.1q37.3 microdeletion and an interstitial microduplication at 2q34q36.1 defined the size of the deletion as 8.42 Mb followed by a single-copy region of 12 Mb and a duplication of 8.57 Mb. This type of chromosomal rearrangement resembles the organization of inv-dup del chromosomes, i.e., inverted duplications contiguous to terminal deletions [10]. These rearrangements originate from a symmetric dicentric chromosome that, after breakage, can generate an inv-dup deletion. In several cases, a single-copy region interposed between the deleted and the duplicated regions has been demonstrated. Inversion heterozygosity in the transmitting parents for this single-copy region has been shown to be a predisposing factor for inv-dup del chromosome formation during meiosis. In all these cases, the duplication immediately proximal to the terminal deletion is inverted, with the single-copy region separating the duplicate copies in an inverted orientation [10,19,20,21,22]. Our FISH results revealed that the duplication in our patient is in tandem and in a ‘direct’ orientation (Figure 3); in addition, neither one of the parents was found to be a carrier of an inversion, which is thought to be a predisposing factor to the rearrangement to occur. Similar configurations for other rearranged chromosomes have hardly been reported [23], and the authors proposed a mechanism in which a double-strand break and illegitimate rejoining of one ‘exposed’ end with a sister chromatid would lead to a recombinant chromatid with both the tandem direct duplication and the terminal deletion. However, in these cases, no single-copy region was found in between the duplication and the deleted regions, suggesting that a different mechanism might have led to the formation of the complex rearrangement in our patient and appears to represent the first case of direct tandem duplication and terminal deletion of chromosome 2. The analysis of the breakpoint intervals on the UCSC Genome Browser reveals that no paralogous segmental duplications map at the breakpoints of the deletion and duplication, suggesting that the dir-dup del (2q) rearrangement in our patient is likely a nonrecurrent rearrangement. Thus, some other mechanism such as NHEJ likely explains this complex rearrangement of chromosome 2q.

Our patient exhibits facial dysmorphism, hypotonia, joint hypermobility, and phenotypic characteristics consistent with those seen in other patients with 2q37 deletion syndrome. Noteworthily, the deleted segment shown in our case is the largest among the annotated cases [3] (Table 1). Given the de novo nature of the variant and the existing data in the scientific literature that attribute a clear pathogenetic role to 2q37.7q37.3 microdeletion, it is reasonable to hypothesize that this variant may underlie the observed clinical presentation. However, our patient’s young age and the lack of instrumental data pertaining to neurological, neuropsychiatric, and cardiological evaluations make it challenging to precisely determine and quantify the contribution of the microduplication 2q34q36.1 to the phenotype. Some cases described in the literature involve microduplication in partially overlapping regions and exhibit clinical features partially comparable with those in our case [5,24,25,26] (Table 2). The neurodevelopmental aspect also requires further evaluation. Nevertheless, considering the noninherited nature of the variant, its size, the number of genes within the duplication interval, and insights from the scientific literature, it is plausible to suggest that this imbalance may have played a contributing role, along with the microdeletion 2q37.7q37.3, in shaping the clinical picture in our case. 

## 5. Conclusions

We report a 15-day-old patient with an 8.42 Mb microdeletion at 2q37.1q37.3 and an 8.57 Mb direct microduplication at 2q34q36.1, comprising 76 and 65 genes, respectively. The de novo nature of the rearrangement, with the peculiar organization of the duplication in tandem and in a direct orientation, as opposed to the typical inverted duplication, renders this case exceptional. Our findings underscore the need to explore chromosomal rearrangements in a subject with phenotypically normal parents, to identify possible mechanisms underlying atypical structural abnormalities and to find new clinical signs that could improve the recognition of syndromes, based on correlations between clinical, molecular, and diagnostic traits. 

## Figures and Tables

**Table 1:** Comparison of clinical findings in previously published cases with 2q37 deletion and those from our patient (adapted from [3]).

Case	P1	P2	P3	P4	P5	P6	P7	P8	P9	Our Patient
Sex	M	F	F	F	M	F	M	F	F	M
Age	30 years	8 years	4 years	12 years	9 years	2 years	18 years	15 years	12 years	9 months
ID	mild	moderate	-	moderate	profound	moderate	severe	moderate	moderate	moderate
Hypotonia	+	+	-	-	+	-	+	+	-	+
Behavioral problems	stereotipies	-	-	ADHD	-	-	autism	aggressivity	laught crises	stereotipies
IUGR	-	-	-	-	-	+	-	-	-	-
Brachydactyly E	+, MT4, 5, AI	+, MT3-5	-	clinodactyly	+, tapering	Al	-	+, MC4, 5	+, MC/MT4, 5	-
Joint hypermobility	+	+	-	-	+	-	-	-	-	+
Low frontal hairline	+/-	high	-	-	+	-	+, widow’s peak	+, widow’s peak	high	-
Frontal bossing	-	+	+	-	+	-	-	-	-	+
Thin/arched evebrows	+, BE	+, BE	-	+, medial sparse	-	+, BE, medial sparse	+, BE	+, BE	+, medial sparse	+, medial sparse
Smooth philtrum	-	+	-	+/-	+	+	+	+	+	+
Thin upper lip	+	+	-	-	+	+	+	+	+	-
Microcephaly	+/-	+/-	-	-	+	++	+/-	+/-	Craniosynostosis	-
Short neck	+	+	+	+	+	+	+	+	+	+
Small/puffy hands/feet	+	-	+	-	-	-	+	+	+/-	+
Deletion size	4.07 Mb	4.07 Mb	5.05 Mb	8.14 Mb	1.84 Mb	2.48 Mb	5.71 Mb	5.71 Mb	4.99 Mb	8.5 Mb
Other CNV	no	no	no	no	Dup 2 (q32.1-q37.3) 42.1 Mb	Dup 2q37.3 1.01 Mb	Dup 9 (q34.11-q34.3) 6.94 Mb	Dup 9 (q34.11-q34.3) 6.94 Mb	Dup 11 (p15.5-p15.4) 1.06 Mb	Dup 2 (q34-q36.1) 8.6 Mb

Age at the last evaluation; + = feature/sign present; - = feature/sign absent; AI = abnormal insertion of toes; BE = bushy eyebrows; ID = global development delay/intellectual disability; IUGR = intrauterine growth restriction; MC = metacarpal bones; MT = metatarsal bones.

**Table 2:** Comparison of clinical findings in previously published patients with isolated 2q trisomy and in our patient (adapted from [5]).

	Fritz et al. [24]	Elbracht et al. [5]	Hermsen et al. [25]	Dahoun-Hadorn and Bretton-Chappius [26]	Our Patient
**Duplicated segment**	2q35-2q37.1	2q35-2q37.3	2q35-2q37.3	2q35-2qter	2q34-2q36.1
**Origin**	de novo	?	de novo	de novo	de novo
	ins 17q25	dup 2q	ins 2p	inv dup 2q	
**Age**	7 years	16 years	9 years	7 years	9 months
**Sex**	M	F	F	M	M
**Birth measurements**	Delivery at term	Delivery at term	Delivery at term	Delivery at term	Delivery at term
Weight	3200 g (50° centile)	3210 g (25–50° centile)	Not reported	4000 g (90° centile)	3780 g (75° centile)
Lenght	51 cm (75° centile)	56 cm (97° centile)	Not reported	Not reported	50 cm (50° centile)
Occipito-frontal Circumference	Not reported	34 cm (25° centile)	Not reported	Not reported	37 cm (25° centile)
**Body measurements at report**					
Weight	19.7 kg (10° centile)	177.6 cm (97°centile)	24.6 kg (10° centile)	26 kg (75–90° centile)	10 kg (75° centile)
Length	120 cm (25° centile)	56.4 cm (97° centile)	131.6 cm (35°centile)	124 cm (50° centile)	73 cm (50–75° centile)
Occipito-frontal circumference	49.9 cm (10–25°centile)	Not reported	50 cm (8° centile)	56 cm (>97° centile)	47 cm (75–90° centile)
**Craniofacial signs**					
Prominent forehead	+	+	+	+	+
Broad nasal bridge	+	+	+	+	-
Overhanging nasal tip	+	+	+	+	-
Thin upper lip	+	+	+	+	+
Retrognathia	+	+	?	+	+
Ears	Large/Low set	Large	Large/Low set	Large/Low set	Large/Low set
**Minor skeletal abnormalities**					
Clinodactyly of fifth finger	?	+	+	+	-
Others	Incomplete syndactyly III/IV of both hands and II/III of both feet				joint hypermobility of ankles and wrists, hypotonia
**Psychomotor retardation**	+ (no further information)	+ (moderate)	+ (reported as moderate)	+	+ (moderate)
**Other CNV**	no	no	no	no	Del 2 (q37.1-q37.3)

Age at the last evaluation; + = feature/sign present; - = feature/sign absent; ? = not evaluated. Bold titles in the first column represent categories of the following specified traits.

## Data Availability

No new data were created or analyzed in this study. Data sharing is not applicable to this article.

## Supplementary Materials

The following supporting information can be downloaded at https://www.mdpi.com/article/10.3390/genes14122194/s1. Figure S1: Karyotype analysis for the proband and the parents. Figure S2: High-resolution SNP array data of the parents of the proband showing no copy number variants on chromosome 2. Table S1: FISH experimental strategy and BAC probes used. Table S2: Dosage sensitive genes embedded in the duplicated and in the deleted regions of our patient. Table S3: Phenotype-associated genes contained in the region duplicated in our patient (https://omim.org/; downloaded on 28 November 2023). Table S4: Phenotype-associated genes contained in the region deleted in our patient (https://omim.org/; downloaded on 28 November 2023). Table S5: Genes comprised in the deleted region and not reported as being associated with any clinical phenotype (https://omim.org/; downloaded on 28 November 2023).
