# Supplementary figures and images for: A Rare Case of Concurrent 2q34q36 Duplication and 2q37 Deletion in a Neonate with Syndromic Features
Source: Genes (Basel). 2023 Dec 10;14(12):2194. doi: 10.3390/genes14122194 (PMC10742419; doi:10.3390/genes14122194)

chromosome 2

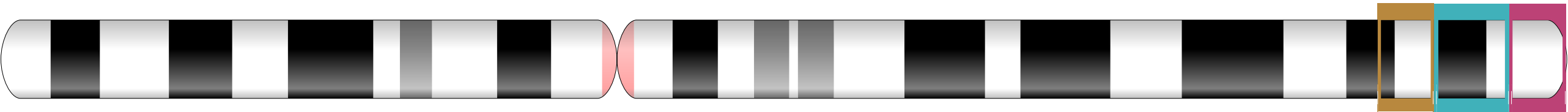

Father PE1713/22

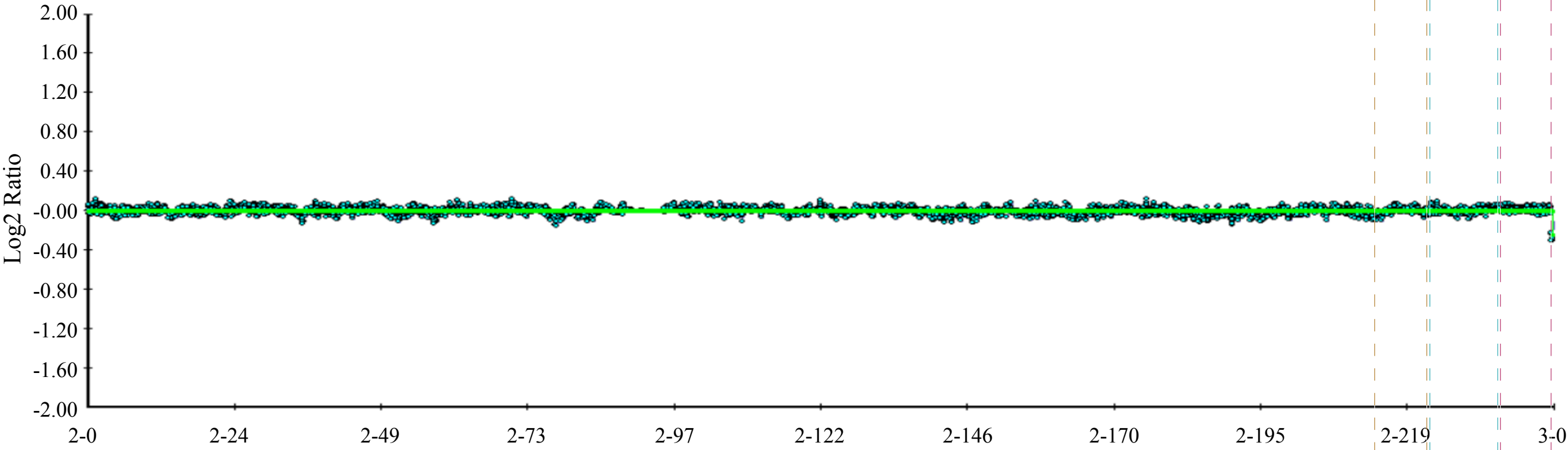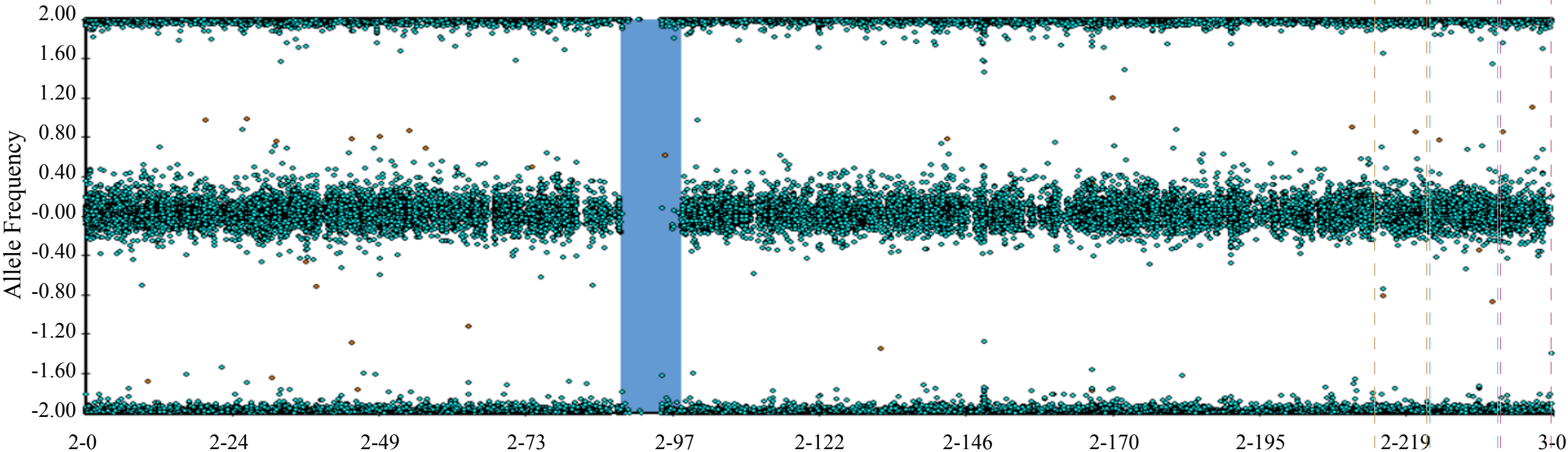

Mother PE1714/22

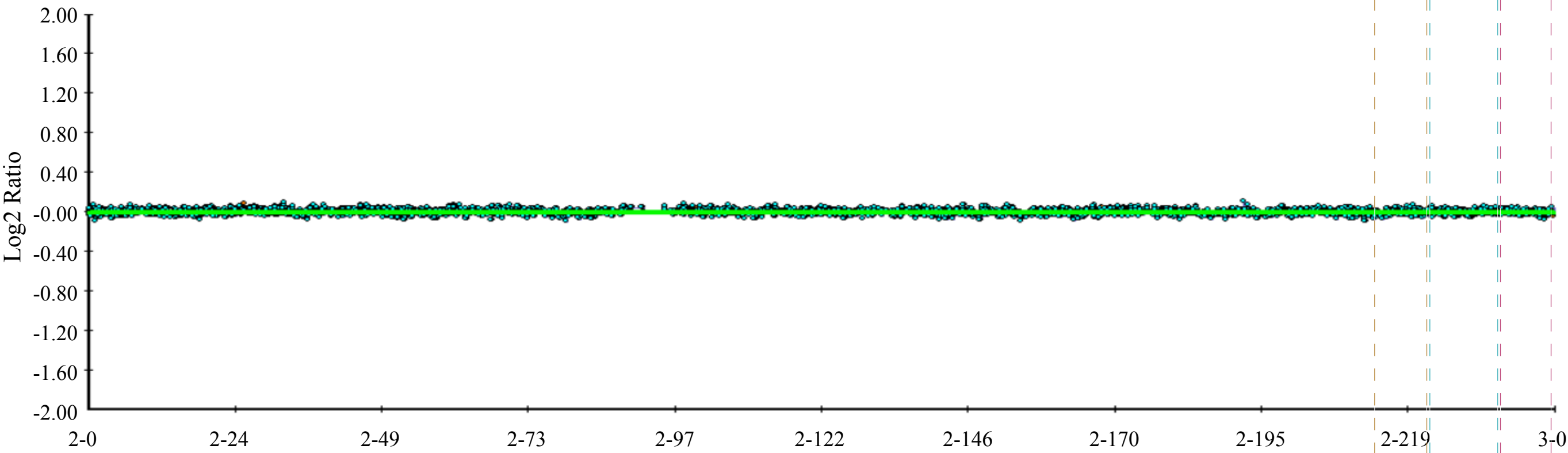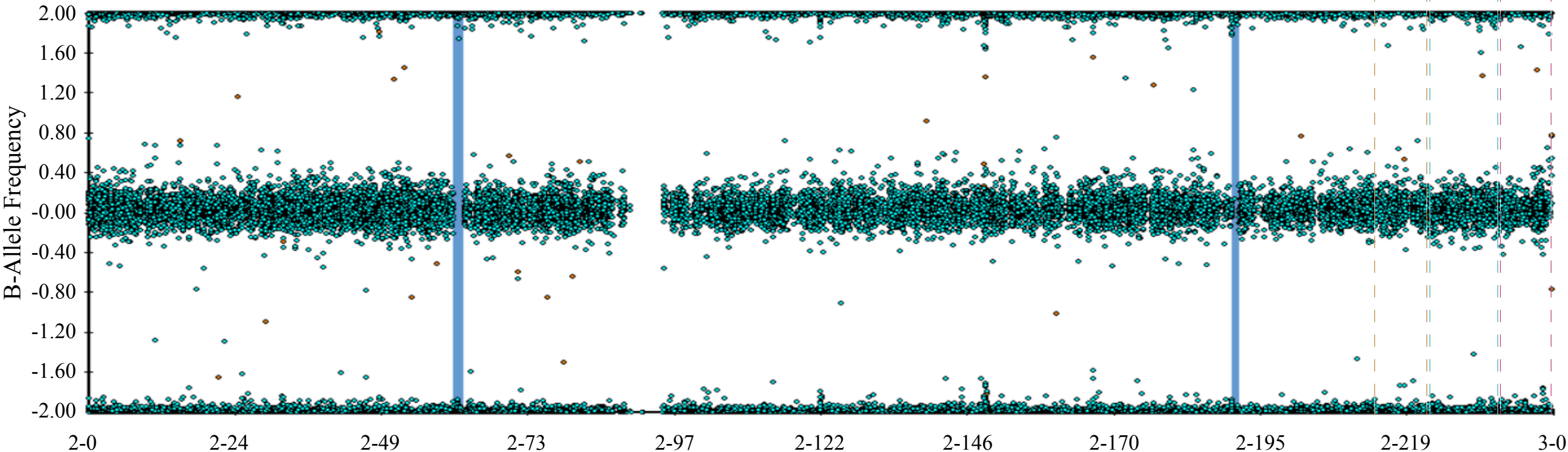

Supplement: Supplementary file 1 [file genes-14-02194-s001.zip › Supplementary Files/Supplementary Figure 2.pdf]

Proband PE1647/22

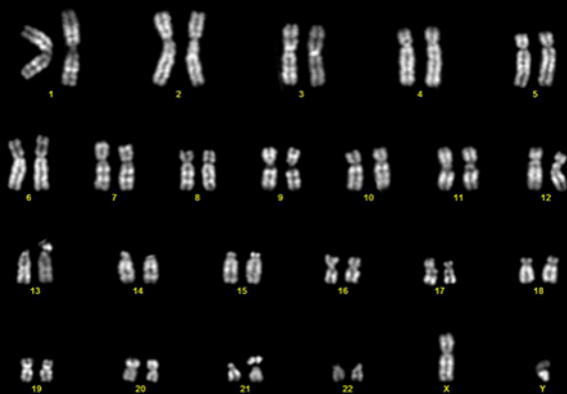

Father PE1713/22

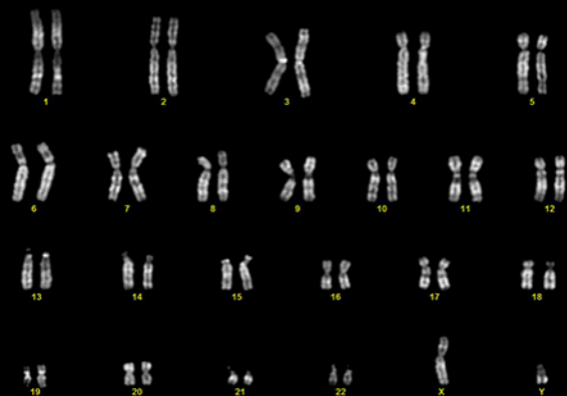

Mother PE1714/22

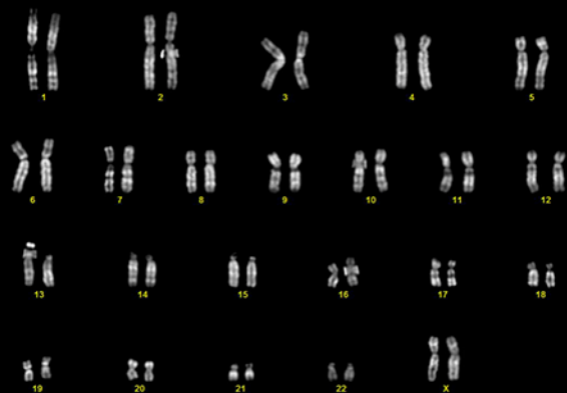

Supplement: Supplementary file 1 [file genes-14-02194-s001.zip › Supplementary Files/Supplementary Figure 1.pdf]
